# *Fusobacterium nucleatum* promotes colorectal cancer liver metastasis via miR-5692a/IL-8 axis by inducing epithelial-mesenchymal transition

**DOI:** 10.1186/s12929-024-01097-4

**Published:** 2025-01-06

**Authors:** Yulong Yu, Han Yin, Bili Wu, Weiheng Zhao, Yuan Wang, Aifeina Aili, Mu Yang, Qianqian Yu, Xianglin Yuan

**Affiliations:** Department of Oncology, Tongji Hospital,Tongji Medical College, Huazhong University of Science and Technology, Wuhan, Hubei China

**Keywords:** Colorectal cancer, *Fusobacterium nucleatum*, Metastasis, IL-8, MicroRNA

## Abstract

**Background:**

The association between the intestinal microbiota and colorectal cancer (CRC) has been extensively studied, with *Fusobacterium nucleatum* (*F. nucleatum*, FN) being found in high abundance in colorectal cancer tissues. Previous research has emphasized the significant role of *F. nucleatum* in the occurrence of CRC. However, the impact of *F. nucleatum* on CRC liver metastasis has not been well understood.

**Methods:**

The effects of *F. nucleatum* on metastasis ability of CRC cell were evaluated in vitro were examined by wound-healing assay and transwell assay. The mouse model of CRC liver metastasis was constructed by spleen injection, and the degree of liver metastasis was assessed by in vivo bioluminescence imaging. The gene expression changes in CRC cells after co-culture with *F. nucleatum* was analyzed through transcriptome sequencing. qRT-PCR and Western Blot assays were performed to validate the expression of related genes and proteins.

**Results:**

The metastasis ability of CRC cells was significantly enhanced after co-culture with *F. nucleatum* in vitro. In the mouse model, *F. nucleatum* also promoted the development of liver metastasis in CRC. Mechanistically, *F. nucleatum* infection increased the expression of IL-8 by downregulated the level of miR-5692a, a regulatory microRNA of IL-8. This led to the activation of the ERK pathway and resulted in the epithelial-mesenchymal transition (EMT) of CRC cells.

**Conclusions:**

Our results suggest that *F. nucleatum* promotes CRC liver metastasis by inducing epithelial-mesenchymal transition through the miR-5692a/IL-8 axis. These findings provide new insights for the prevention and treatment of colorectal cancer liver metastasis.

**Supplementary Information:**

The online version contains supplementary material available at 10.1186/s12929-024-01097-4.

## Introduction

Colorectal cancer is one of the most prevalent cancers worldwide, with about 1.9 million new cases diagnosed annually [1, 2]. Metastasis of CRC continues to pose a significant challenge following curative treatment, representing the primary factor contributing to mortality associated with CRC [3]. Approximately half of CRC patients will experience liver metastasis, and the 5-year survival rate for patients with liver metastases stands at a mere 14–30%^4–6^. Surgical resection and chemotherapy represent the therapeutic interventions that provide extended survival prospects for patients diagnosed with colorectal liver metastases. Nevertheless, a staggering 80% of these cases are deemed inoperable due to the extensive number and size of metastases, as well as the involvement of multiple liver segments [7]. Therefore, it is imperative to comprehensively investigate the underlying mechanisms driving CRC metastasis.

A primary hallmark of CRC is dysbiosis of the gut microbiota, which is defined by a reduction in the diversity of microorganisms and an increase in pathobionts responsible for cancer [8]. Accumulating evidence substantiates the impact of cancer-associated microbiota on the development and progression of cancer, particularly in the case of colorectal cancer [9, 10]. The gut microbiota can produce genotoxins to damage the DNA of colonic epithelial cells and activate oncogenic signaling pathways such as the Wnt–β-catenin signaling pathway [11, 12]. Moreover, bacteria-driven inflammation triggers the secretion of chemokines to induce CRC cells to acquire stemness and boost metastasis formation [13]. *F. nucleatum*, an anaerobic opportunistic pathogen, is abundant in both the fecal matter and tumor tissues of patients with colorectal cancer and is linked to tumor invasion and distant metastasis [14, 15]. *F. nucleatum* accompanies primary tumor cells during the process of metastasis, contributing to the colonization of tissues in distant locations [16]. However, the mechanisms underlying the role of *F. nucleatum* in CRC liver metastasis need to be fully elucidated.

There is growing evidence that chemokines triggered by bacteria are implicated in various tumorigenesis processes [17]. Interleukin-8 (IL-8), a member of the chemokine family, has been found to play a role in the proliferation, migration, and invasion of cancer cells [18, 19]. Previous studies have shown that infection with *F. nucleatum* can lead to an increase in IL-8 expression [20]. However, whether *F. nucleatum* influences IL-8 to promote CRC liver metastasis remains uncertain. The mechanisms underlying the regulation of IL-8 by *F. nucleatum* are also unclear.

In this study, we aimed to investigate the association between *F. nucleatum* infection and CRC liver metastasis, as well as the role of IL-8 in progression. The results of this study will establish a strong foundation for targeting *F. nucleatum* and IL-8 in the treatment of CRC liver metastasis.

## Materials and methods

### Bacterial strain and culture conditions

The *F. nucleatum* strain (ATCC 25586) was obtained from the China General Microbiological Culture Collection Center and cultured anaerobically (MGC, AnaeroPack) in brain heart infusion (Landbridge, CM917B) broth medium at 37 °C.

### Cell Culture

The human CRC cell lines (HT29, SW480) and the mouse CRC cell line CT26 were purchased from the Cell Bank of the Chinese Academy of Sciences (Shanghai, China) and preserved in liquid nitrogen. Three types CRC cell lines were cultured in high-glucose DMEM (Hyclone, SH30243) or RPMI 1640 (Hyclone, SH30027) at 37℃ with 5% CO2 in incubator, respectively. The media were supplemented with 10% fetal bovine serum (FBS, Gibco). *F. nucleatum* and CRC cells were co-cultured for 24 h according to MOI = 100:1.

Recombinant human IL-8(MCE, HY-P7224) was dissolved in PBS with 0.1%BSA at a concentration of 100ng/ml. Reparixin (an inhibitor of CXCR1/2, MCE, HY-15251), MK2206(an inhibitor of AKT, MCE, HY-10358), U0126(an inhibitor of ERK, MCE, HY-12031), SB203580(an inhibitor of p38, MCE, HY-10256), and SP600125(an inhibitor of JNK, MCE, HY-12041) were purchased from Med Chem Express.

### Animal experiments

For liver metastasis model, male BALB/C mice of 8-week-old were randomly divided into four groups (groups: control, *F. nucleatum*, Reparixin, *F. nucleatum* + Reparixin, 5mice/group). The mice were anesthetized and given injections of 1×10^6^ CT26 cells or *F. nucleatum* infected CT26 cells into the spleen. Reparixin and *F. nucleatum* + Reparixin groups started intraperitoneal injection of Reparixin (30 mg/kg) every three days on the third day after modeling. All animal experiments were conducted according to the Guidelines of the Institutional Animal Care and Use Committee and was approved by the Animal Ethics Committee of Tongji Hospital.

### GMrepo database analysis

The GMrepo is a curated resource of consistently annotated human gut metagenomes. The relative abundances of *F. nucleatum* in the stool of healthy and CRC patients were extracted from GMrepo database (https://gmrepo.humangut.info). By consulting the data from relative publications, the data’s quality was evaluated.

### Cell transfection

miR-5692a mimics, inhibitors, and negative control were obtained from RiboBio (Guangzhou, China). The transfection of miRNA was performed with riboFECTTM CP Transfection Kit (RiboBio, C10511-05). The short hairpin RNAs specific to ZEB1 was obtained from Genechem and was transfected into CRC cells according to the manufacturer’s protocols.

### Dual-luciferase assay

Dual-luciferase assay was conducted to confirm the regulatory effect of miR-5692a on IL-8 expression. Luciferase reporter plasmid vectors (RiboBio) with wild type or mutant 3′-untranslated region of IL-8 was co-transfected respectively with miR-5692a mimics or inhibitor into CRC cells. The luciferase activity was detected after 48 h incubation using Dual-Luciferase Reporter Assay System (Promega, E1910).

### RNA extraction and quantitative real-time PCR

Total RNA extraction from CRC lines using RNAiso reagent (Vazyme, R701) following the manufacturer’s instructions. HiScript RT SuperMix reagent kit (Vazyme, R323) was used for complementary DNA synthesis. Real-time PCR was performed using ChamQ Universal SYBR Master Mix kit (Vazyme, Q711) according to the protocol and *GAPDH* mRNA was used as a reference gene. The 2^(−ΔΔCt)^ method was applied for calculating the relative mRNA. The primers sequences used in this study are listed in Table S1.

### RNA sequencing

RNA sequencing was conducted by Novogene (Beijing, China), with the expression levels of all samples represented as RPKM (Reads Per Kilobase per Million). Differentially expressed genes were visualized using the “pheatmap” and “ggplot2” R packages. For KEGG enrichment analysis, a significance threshold of *p* < 0.05 was applied to identify enriched gene sets using the “clusterProfiler” R package.

### Wound healing assay

Cells were seeded on the 6-wells plates and cultured to 90% confluence. Cells were treated with 10 µg/mL mitomycin C for 2 h prior to wounding in order to impair proliferation. Then the cells were scraped with a sterile pipette tip to generate a vertical scratch(wound) and grown in the serum-free medium. Subsequently, the scratch was photographed under microscope at 0 h and 48 h, respectively.

### Colony formation assay

The Colony Formation assay was performed on standard tissue culture-treated 6-well plates without any surface coating. A total of 1×10^3^ CRC cells per well were seeded in 6-well plates and cultured in DMEM supplemented with 10% FBS and 1% penicillin-streptomycin. Fourteen days after plating, the cells were fixed with 4% paraformaldehyde and stained with 0.05% crystal violet. The number of colonies, representing the proliferation rate, was counted.

###  EdU incorporation assay

To perform the EdU incorporation assay, 5×10^3^ CRC cells per well were seeded into 96-well plates and cultured overnight. The EdU Cell Proliferation Image Kit (Abbkine, Wuhan, China) was used following the manufacturer’s instructions. Briefly, cells were incubated with 100 µl of 10 µM EdU solution for 2 h. After incubation, the cells were washed with PBS, fixed with 4% paraformaldehyde, and permeabilized with 0.5% Triton X-100. The cells were then stained with 100 µl of 1X AbFluor 545 Azide solution for 30 min, followed by nuclear staining with 1X Hoechst 33,342 solution. Images were captured using a Leica fluorescence microscope (Germany), and the number of positive cells was counted.

### Cell invasion assays

A 24-well transwell plate with 8-µm pore size (Corning, 3422) was carried out for transwell assays. For cell invasion assays, the upper chambers were coated with matrigel. Starving cells for 24 h in advance, then cells were seeded into the upper chambers with a cell density of 5×10^4^/well. Resuspend cells in serum-free medium or conditioned medium. The lower chambers were added with 30% serum-containing medium. After then incubation for 24–48 h, the transwell insert chamber membrane was fixed with 4% paraformaldehyde for 15 min and stained with 0.1% crystal violet for 15 min. Cells that did not successfully invade through the membrane were mechanically removed with a cotton swab. Cells invading through the membrane which on the bottom surface of the membrane were captured and counted at the random fields using a microscope.

### Western blot analysis

Total protein was lysed by the RIPA buffer (Servicebio, G2002) mixed with 1% protease inhibitor and 1% phosphorylase inhibitor. The extracted protein was fractionated with 10% sodium dodecyl sulfate polyacrylamide gel electrophoresis, and then transferred to polyvinylidene difluoride membrane (Merck, C3117). The membranes were blocked with 5% milk diluted with TBST (for 1 h at room temperature. Then, the blots were incubated with specific primary antibody at 4℃ overnight. The GAPDH protein level on the same membrane were used for the loading control. The membranes were washed three times by TBST, then incubated with the horseradish peroxidase-labeled secondary antibody for 1 h at 37 ℃. Proteins were visualized with an enhanced chemiluminescence reagent (Thermofischer, 34579) using the system (Bio-Rad). The primary antibodies used are listed in Table S2.

### Immunohistochemistry (IHC) analysis

The paraffin-embedded tissue samples were sliced into 4 μm thick sections, and various IHC assays were performed according to the manufacturer’s instructions. The relative expression was scored according to the staining scope and intensity. Specifically, the staining scope was: 1 (0–25%); 2 (25–50%); 3 (50–75%); and 4 (75–100%), and the staining intensity was scored as 0 (negative); 1 (weakly positive); 2 (moderately positive); and 3 (strongly positive). The overall score was defined by multiplying the staining scope by the staining intensity score.

## Results

### ***F. nucleatum ***is enriched in CRC patients and promotes proliferation, migration and invasion of CRC cells

**Fig. 1:**
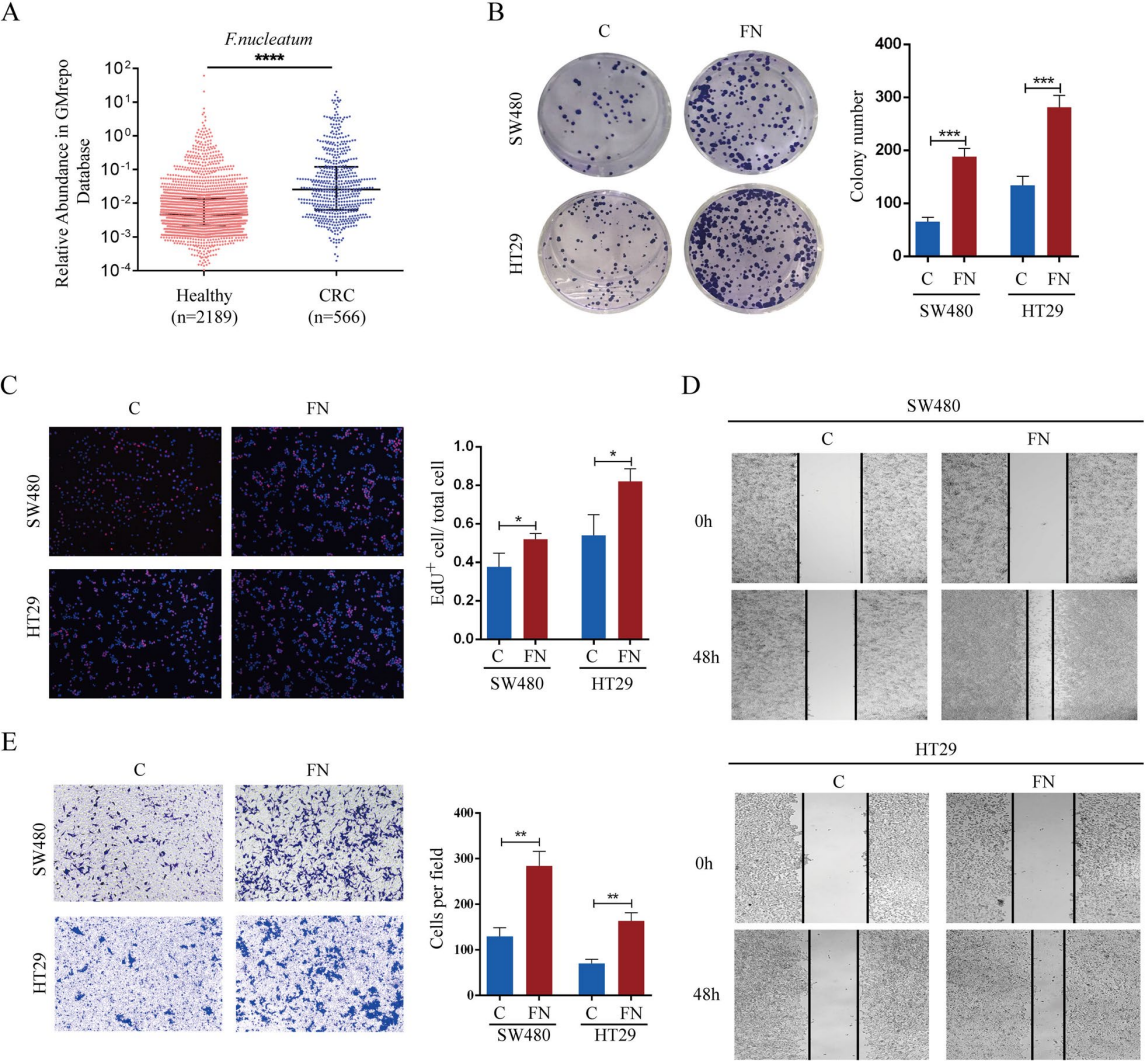
*F. nucleatum* is enriched in CRC patients and promotes proliferation, migration and invasion of CRC cells. **A** *F. nucleatum* prevalence in the feces of healthy individuals and CRC patients in the GMrepo database (Mann-Whitney test). **B, C** *F. nucleatum* promoted proliferation of SW480 and HT29 cells in colony formation (**B**) and EdU assays (**C**). **D** *F. nucleatum* accelerated wound healing of SW480 and HT29 cells compared with the control. **E** The invasion ability of SW480 and HT29 cells was detected by transwell assay

To investigate the disparity in the gut microbiota between CRC patients and healthy individuals, we conducted a comparative analysis of the abundance of 219 marker taxa in the GMrepo Database. The marker taxa with linear discriminant analysis (LDA) scores greater than 2 or less than − 2 are presented in Figure S1A. Notably, the abundance of *F. nucleatum* significantly increased in CRC patients (Fig. 1A), suggesting a potential influential role of *F. nucleatum* in the progression of CRC. To ascertain whether *F. nucleatum* could facilitate the growth of CRC cells, SW480 or HT29 cells were preincubated with *F. nucleatum* for 24 h and subsequently subjected to colony formation and EdU assays. Consistent with our expectations, the presence of *F. nucleatum* resulted in a significant increase in the number of colonies and EdU-positive cells compared to those in the control group (Fig. 1B, C). These findings suggest that *F. nucleatum* enhances the proliferation of CRC cells. Furthermore, wound healing and transwell assays were conducted to assess the impact of *F. nucleatum* on the motility of CRC cells. The results revealed that, compared with control cells, SW480 or HT29 cells exposed to *F. nucleatum* exhibited faster wound closure and a greater number of invading cells (Fig. 1D, E). Taken together, these findings suggest that *F. nucleatum* enhances the proliferation, migration and invasion of CRC cells.

### The expression of IL-8 increases in ***F. nucleatum ***treated CRC cells and is associated with the metastasis of CRC

**Fig. 2:**
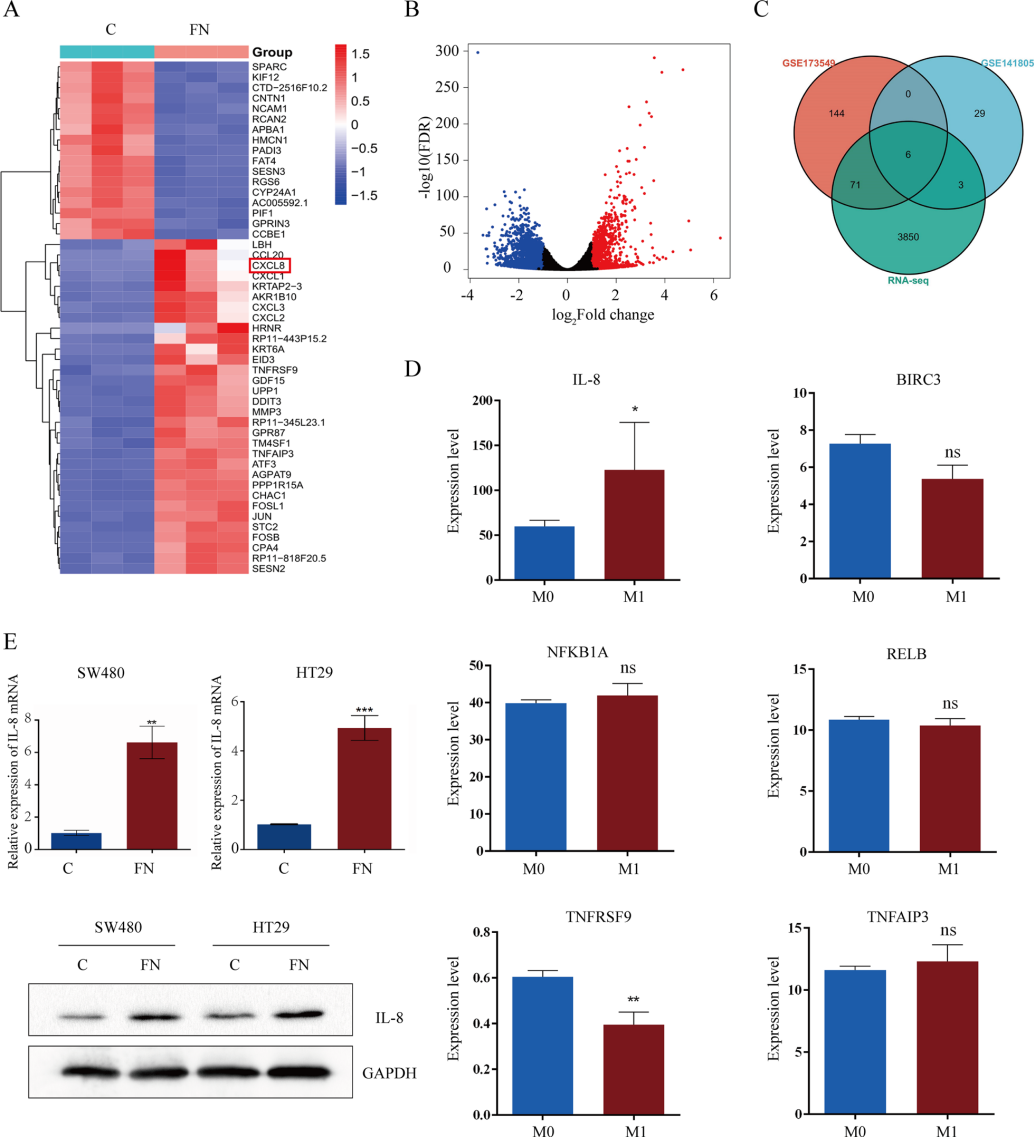
The expression of IL-8 increases in *F. nucleatum* treated CRC cells and is associated with metastasis of CRC. **A, B** The Heat map **A** and Volcano map **B** were used to depict the differential gene expression patterns between *F. nucleatum*-treated and PBS-treated HT29 cells by RNA-seq (*n* = 3, log2 fold change > 1, *P* < 0.05). CXCL8, the gene encoding IL-8, is indicated in the heat map. **C** Overlap of differential gene from our RNA-seq data, GSE141805 and GSE173549 dataset. **D** Relative expression of IL-8, BIRC3, NFKBIA, RELB, TNFRSF9, and TNFAIP3 between M0 and M1 CRC patients in TCGA database. **E** qRT-PCR and western blot analysis of IL-8 mRNA and protein level in SW480 and HT29 cells after *F. nucleatum* infection. The Western blot in bottom panel is quantified in Supplementary Figure S2A

To determine the mechanism underlying the interaction between *F. nucleatum* and CRC cells, we cocultured HT29 cells with *F. nucleatum* for 24 h and conducted transcriptome sequencing (RNA-Seq) analysis. The differential expression patterns of the genes were visualized using a heatmap (Fig. 2A). A total of 3930 Differentially Expressed Genes (DEGs) were identified by the adjusted *P* value and fold change (FC) value (Fig. 2B). We further analyzed the data in the GSE173549 and GSE141805 datasets and combined them with our RNA-Seq data. Six DEGs coexisted in the three datasets (Fig. 2C). To determine which gene is related to the metastasis of CRC, we compared the expression of the 6 genes between M0 CRC patients and M1 CRC patients in The Cancer Genome Atlas (TCGA) database. Among them, only the IL-8 concentration increased in metastatic patients (Fig. 2D). qRT‒PCR and western blot assays confirmed the overexpression of IL-8 in CRC cells after coculture with *F. nucleatum* (Fig. 2E). Thus, *F. nucleatum* may promote CRC metastasis by increasing the expression of IL-8.

### ***F. nucleatum*** promotes metastasis and EMT phenotypes of CRC cells in a IL-8 dependent manner

**Fig. 3:**
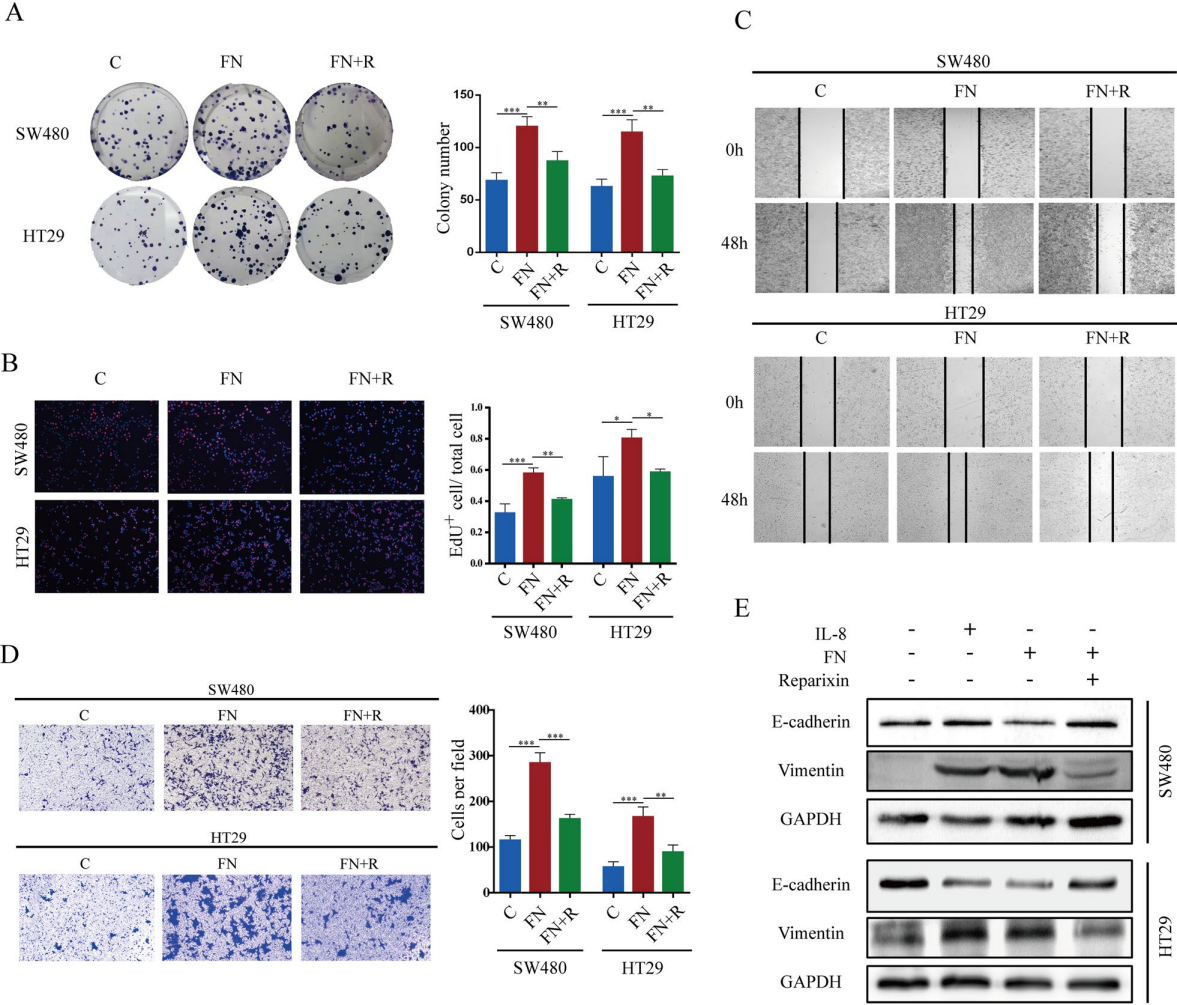
*F. nucleatum* promotes metastasis and EMT phenotypes of CRC cells in an IL-8 dependent manner. **A, B** colony formation and EdU assays showed the proliferation capacity of CRC cells treated with *F. nucleatum* and/or Reparixin. **C, D** wound healing and transwell analysis were performed to determine the migration and invasion capacity of CRC cells. **E** western blot was performed to evaluate the expression of EMT marker in SW480 and HT29 cells after treated with *F. nucleatum* and Reparixin. The Western blot is quantified in Supplementary Figure S2B

To confirm that *F. nucleatum* affects CRC cells via IL-8, we used Reparixin to inhibit the function of the IL-8 receptor. Reparixin significantly limited the proliferation, migration and invasion of CRC cells after *F. nucleatum* treatment (Fig. 3A–D). Epithelial-Mesenchymal Transition is a critical process in cancer metastasis [21]. During this process, epithelial cells lose their characteristic features and acquire traits typical of mesenchymal cells, which enhances their motility and invasion capacity. We hypothesized that *F. nucleatum* promotes CRC cell migration by enhancing the EMT phenotype. To confirm our hypothesis, we conducted western blot to examine the changes in EMT-related genes in SW480 or HT29 cells. We observed that *F. nucleatum* led to elevated expression of the mesenchymal marker vimentin and decreased expression of the epithelial marker E-cadherin. Furthermore, Reparixin suppressed EMT in *F. nucleatum*-treated CRC cells (Fig. 3E). Collectively, these results suggest that *F. nucleatum* can promote CRC cell proliferation and migration by enhancing EMT phenotypes in an IL-8-dependent manner.

### ***F. nucleatum ***promotes liver metastasis of CRC cells in vivo

**Fig. 4:**
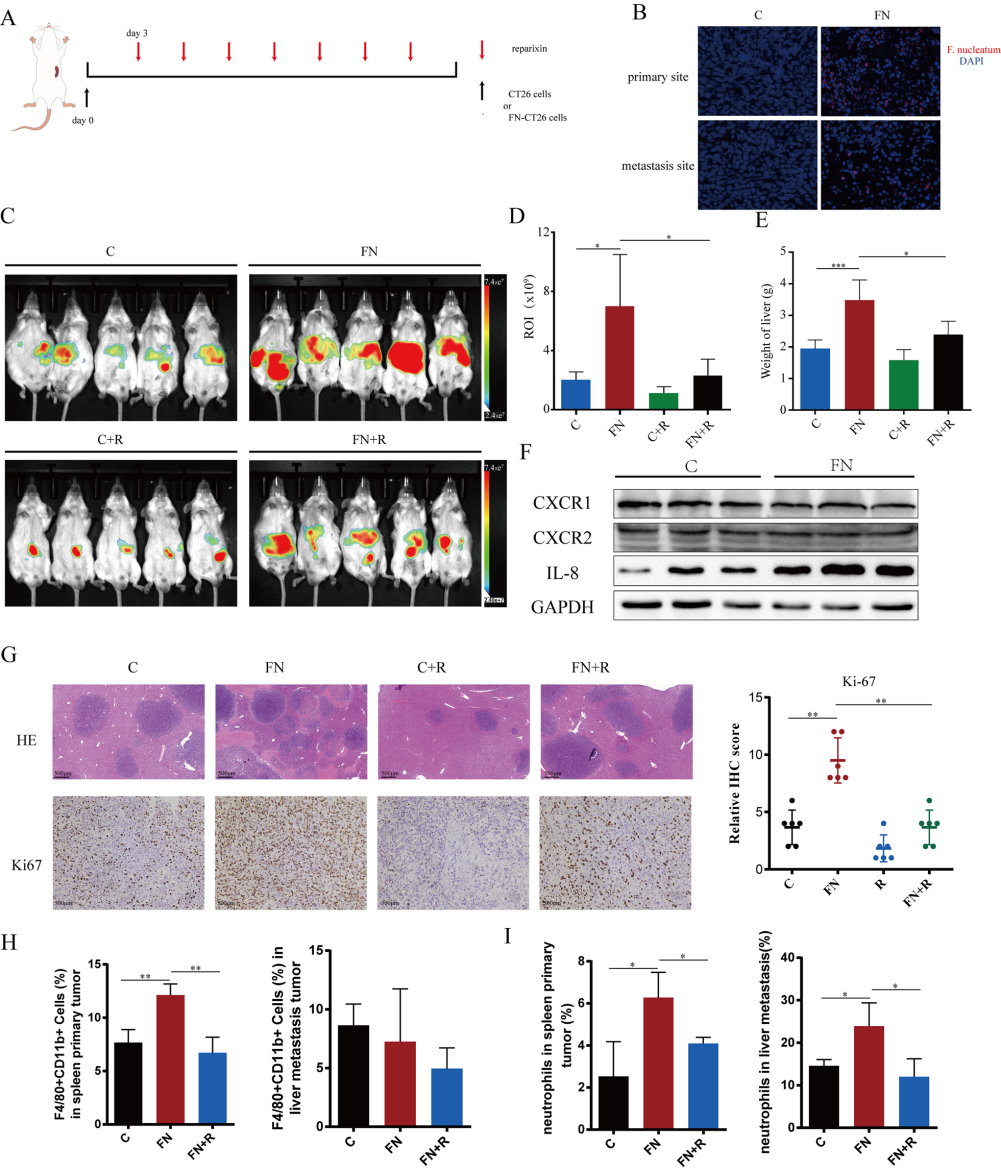
*F. nucleatum* promotes metastasis of CRC cells in vivo. **A** Pretreated with *F. nucleatum* or PBS for 24 h, CT26 cells were injected into mice via spleen (*n* = 5). **B** Representative images from FISH analysis showing *F. nucleatum* presence (red) of primary tumors in spleen and metastases site in liver. **C–E** The liver metastases were assessed by in vivo bioluminescence imaging (**C, D**) and the weight of liver (**E**). **F** IL-8 expression and CXCR1/2 expression by the tumor cells in vivo. The Western blot is quantified in Supplementary Figure S2C. **G** Representative images of H&E and IHC staining of Ki67 in liver metastases of mice. **H** The frequencies of macrophages in spleen primary tumor and liver metastasis. **I** The frequencies of neutrophils (CD45^+^CD11b^+^Ly6G^+^) in spleen primary tumor and liver metastasis

CT26-Luc cells or *F. nucleatum*-treated CT26-Luc cells were injected into the spleen of BALB/c mice to establish a liver metastasis model. To block IL-8 function, Reparixin was administered by intraperitoneal injection on the third day after the model was established (Fig. 4A). In the *F. nucleatum* coculture group, we observed *F. nucleatum* presence in both primary and metastasized tumors. Conversely, the control group showed no presence of *F. nucleatum*(Fig. 4B). The liver metastases of CT26-Luc cells were tracked using an in vivo optical imaging system. The results showed that liver fluorescence intensity was greater in the *F. nucleatum*-treated group than in the control group, indicating that more CT26-Luc cells metastasized to the liver. Furthermore, Reparixin inhibited the *F. nucleatum*-mediated promotion of CT26-Luc cell metastasis in vivo, suggesting that *F. nucleatum* promotes CRC liver metastasis in vivo through IL-8 (Fig. 4C, D). The liver weight results were consistent with the above conclusion (Fig. 4E). We confirmed that in the *F. nucleatum*-treated group, IL-8’s mouse homolog expression was elevated in vivo, CXCR1/2 expression remained unchanged (Fig. 4F). The expression of ki-67 and Vimentin was significantly higher in the *F. nucleatum* coculture group than in the control group, while E-cadherin reduced (Fig. 4G, Figure S3). IL-8 is known as a chemoattractant for immune cells, including neutrophils and macrophages. To explore this, we conducted flow cytometry to assess immune cell infiltration. Our findings revealed an increased infiltration of macrophages, particularly M2-polarized macrophages, at the injection site in the F. nucleatum-treated group, which was reduced upon treatment with Reparixin. However, this effect was not observed at the metastasis site in the liver (Fig. 4H). Additionally, we observed a significant accumulation of neutrophils at both the injection and metastasis sites in the F. nucleatum-treated group, which was also reversed by Reparixin treatment (Fig. 4I). These results suggest that F. nucleatum may promote tumor progression and metastasis by enhancing IL-8-mediated infiltration of macrophages and neutrophils, thereby altering the tumor microenvironment and driving the metastasis of CRC cells in an IL-8-dependent manner.

### ***F.nucleatum ***increases the expression of IL-8 though miR-5692a

**Fig. 5:**
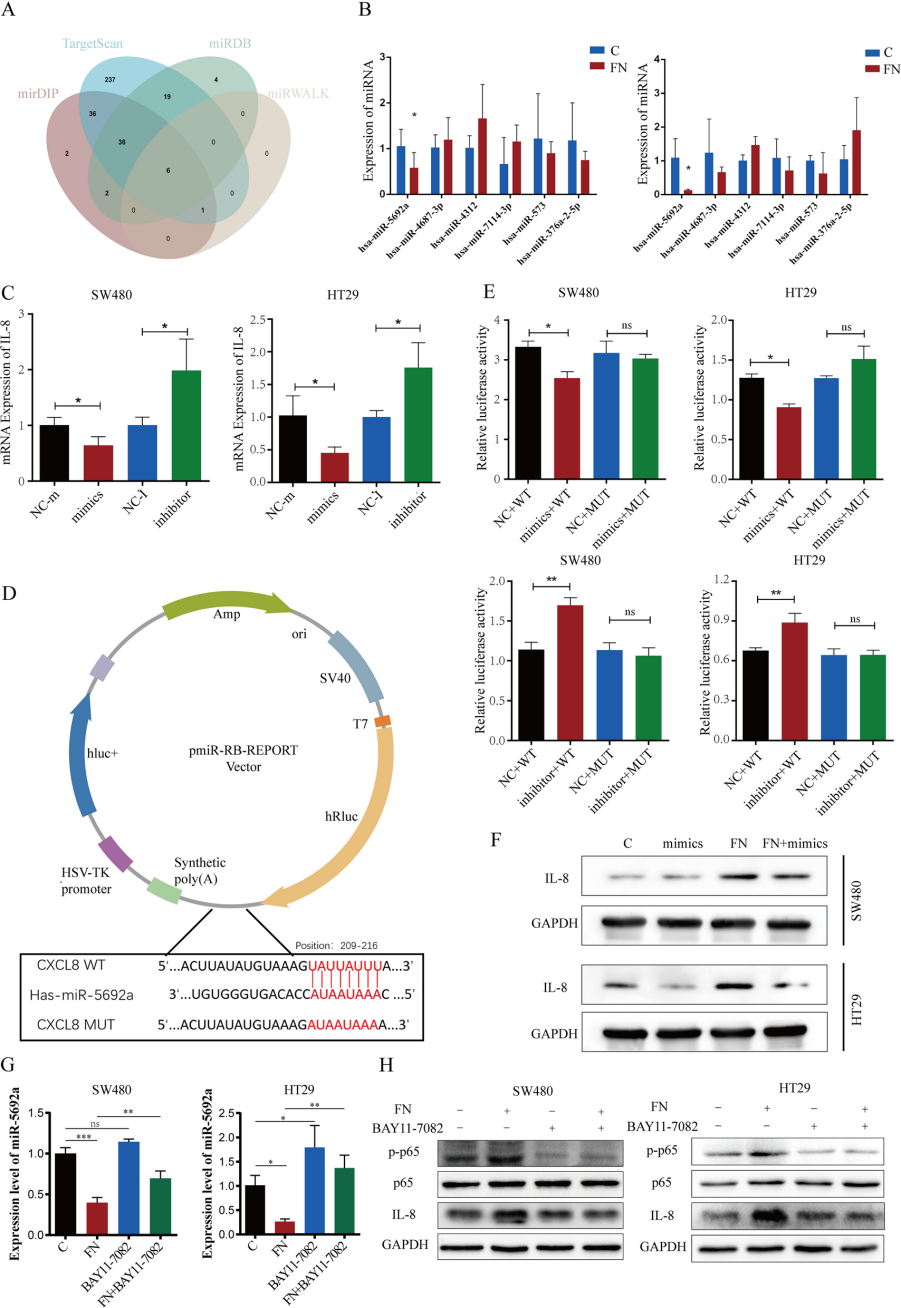
*F. nucleatum* increases the expression of IL-8 though miR-5692a. **A** Overlap of the predicted miRNAs targeting IL-8 mRNA from four different websites. **B** qRT-PCR analysis the expression of candidate miRNAs binding to IL-8 in CRC cells. **C** qRT-PCR analysis of IL-8 mRNA level after miR-5692a overexpression (mimics) or downregulation (inhibitor). **D** Schematic image of luciferase reporter plasmids with miR-5692a binding site of IL-8. **E** SW480 and HT29 cells were transfected with wild type (WT) or mutant (MUT) luciferase reporter plasmids with miR-5692a mimics or inhibitor. Forty-eight hours after transfection, cells were subjected to dual-luciferase assay. **F** The protein level of IL-8 was evaluated after treated with *F. nucleatum* and/or miR-5692a mimics. The Western blot is quantified in Supplementary Figure S2D. **G** The expression of miR-5692a in CRC cells after infected with F. nucleatum and/or NF-κB pathway inhibitor Bay11-7082 (20 µM). **H** The expression of p-p65, p65 and IL-8 in CRC cells after infected with F. nucleatum and/or NF-κB pathway inhibitor Bay11-7082 (20 µM)

miRNAs can bind to the 3’UTR of target genes to regulate target gene expression and play a key role in the development of cancers. We used mirDIP, TargetScan, miRDB and miRWALK to predict miRNAs that potentially bind to the IL-8 mRNA and identified six candidate miRNAs (Fig. 5A). To validate the predicted results and detect the expression of candidate miRNAs, SW480 and HT29 cells were infected with *F. nucleatum*. qRT‒PCR revealed that miR-5692a expression markedly decreased after *F. nucleatum* treatment among all potential miRNA (Fig. 5B). Then, the miR-5692a mimics and miR-5692a inhibitor were transfected into SW480 and HT29 cells. qRT-PCR demonstrated that overexpression of miR-5692a downregulated and inhibition of miR-5692a upregulated the expression of IL-8 (Fig. 5C). To validate whether miR-5692a directly binds to the mRNA of IL-8, we generated wild-type (WT) or mutated (MUT) dual-luciferase reporter plasmids (Fig. 5D). SW480 and HT29 cells were transfected with dual-luciferase reporter plasmids with miR-5692a mimics or miR-5692a inhibitors. miR-5692a mimics significantly suppressed and miR-5692a inhibitors enhanced the luciferase activity of WT plasmids compared with MUT plasmids (Fig. 5E). The miR-5692a mimics downregulated the expression of IL-8, which was upregulated in CRC cells after incubation with *F. nucleatum* (Fig. 5F). Colony formation, EdU, wound healing and Transwell assays revealed that miR-5692a mimics reversed the promotion of cell proliferation, migration and invasion in *F. nucleatum*-treated CRC cells (Figure S1B–E). As a result, we deduced that *F. nucleatum* promotes CRC proliferation and metastasis through the miR-5692a/IL-8 axis.

Previous studies have demonstrated that *F. nucleatum* can activate the NF-κB pathway in various cancer cells [22, 23]. To explore whether F. nucleatum influences miR-5692a and IL-8 expression through the NF-κB pathway, we assessed the activation of NF-κB. The results showed significant NF-κB activation in CRC cells treated with F. nucleatum. When the NF-κB inhibitor BAY11-7082 was applied, the F. nucleatum-induced down-regulation of miRNA-5692a and up-regulation of IL-8 were notably reduced (Fig. 5G, H). This indicates that F. nucleatum may partially regulate miRNA-5692a and IL-8 expression via the NF-κB pathway.

### ***F. nucleatum***-induced IL-8 activates ERK pathway

**Fig. 6:**
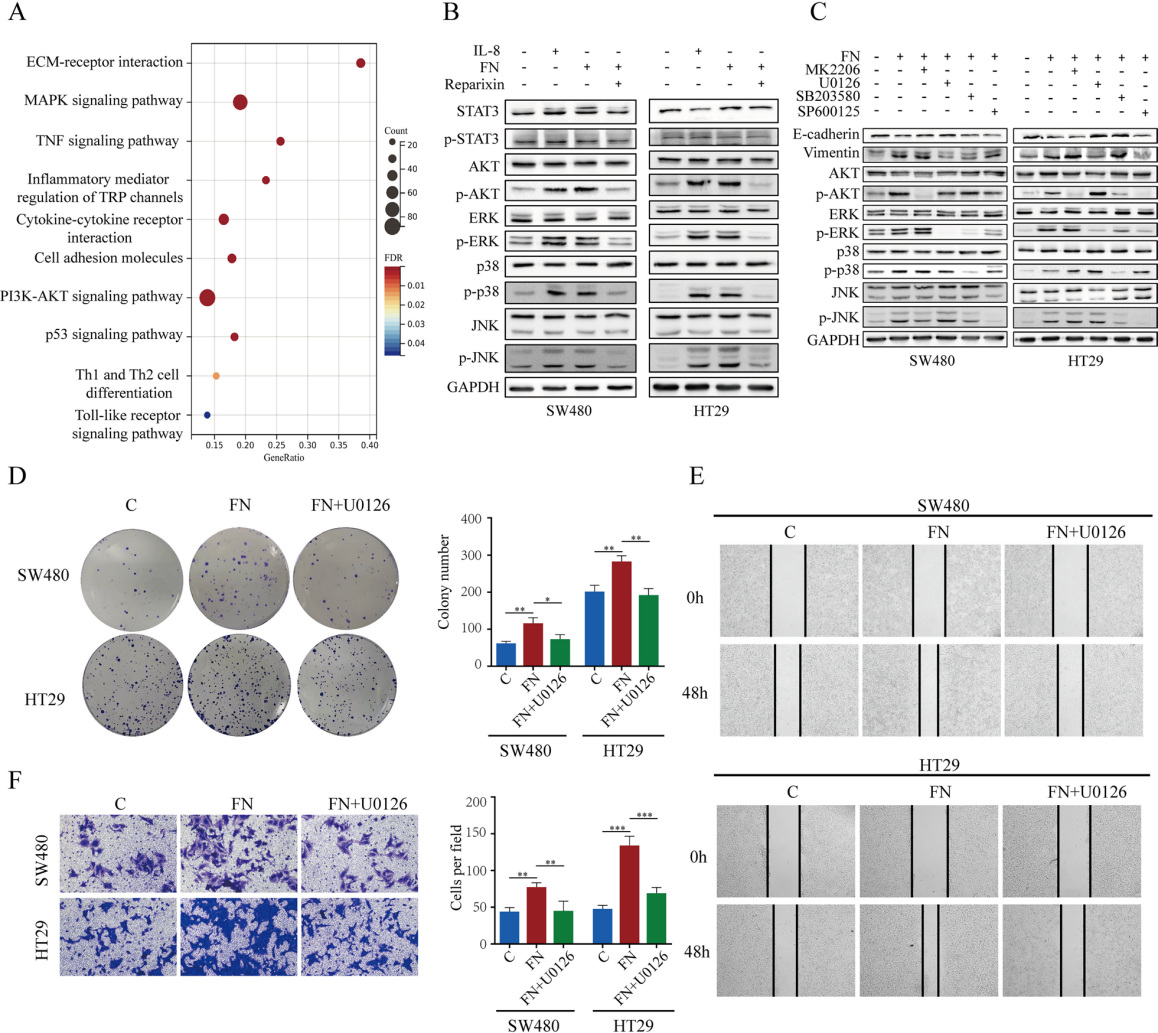
*F. nucleatum* induced IL-8 activates ERK pathway. **A** KEGG enrichment analysis identified the relative signal pathway in *F. nucleatum*-treated HT29 cells. **B** After infected with *F. nucleatum* and/or Reparixin, the protein level of AKT, ERK, p38 AND JNK were measured by western blot. The Western blot is quantified in Supplementary Figure S2E. **C** After infected with *F. nucleatum* and/or small molecule inhibitors, the expression of EMT marker were tested by western blot. MK2206, an inhibitor of AKT; U0126, an inhibitor of ERK; SB203580, an inhibitor of p38; SP600125, an inhibitor of JNK. **D** colony formation showed the proliferation capacity of CRC cells treated with *F. nucleatum* and/or U0126. **E, F** wound healing and transwell analysis showed the migration and invasion capacity of CRC cells treated with *F. nucleatum* and/or U0126

To assess the significant pathways changed in *F. nucleatum*-treated CRC cells, Kyoto Encyclopedia of Genes and Genomes (KEGG) analysis was performed, and the significant pathways were selected (Fig. 6A). The MAPK and PI3K-AKT pathways were significantly activated in *F. nucleatum*-treated CRC cells. The phosphorylation levels of STAT3, AKT, ERK, P38, and JNK in SW480 and HT29 cells were assessed via western blot (Fig. 6B). The results showed that IL-8 and *F. nucleatum*-treated CRC cells had increased AKT, ERK, P38 and JNK activity. Reparixin can block the activating effects of *F. nucleatum* on AKT, ERK, P38, and JNK. To determine which of these proteins is involved in EMT, we used inhibitors of AKT, ERK, P38, and JNK together with *F. nucleatum*. Only the inhibitor of ERK rescued *F. nucleatum*-induced EMT in CRC cells (Fig. 6C). These results indicated that *F. nucleatum*-induced IL-8 promoted EMT in CRC cells through the ERK pathway. The proliferation and metastasis ability of *F. nucleatum*-cocultured CRC cells were also inhibited by the ERK inhibitor (Fig. 6D–F). Since HT29 contains a BRAF mutation, and SW480 and CT26 harbor KRAS mutations, we further conducted in vitro validation using CACO-2 cells (Figure S4A-D) and in vivo validation with MC38 cells (Figure S4E), both of which do not contain BRAF or KRAS mutations.

### ***F. nucleatum***-induced IL-8 promotes CRC metastasis through ERK/ZEB1 axis

**Fig. 7:**
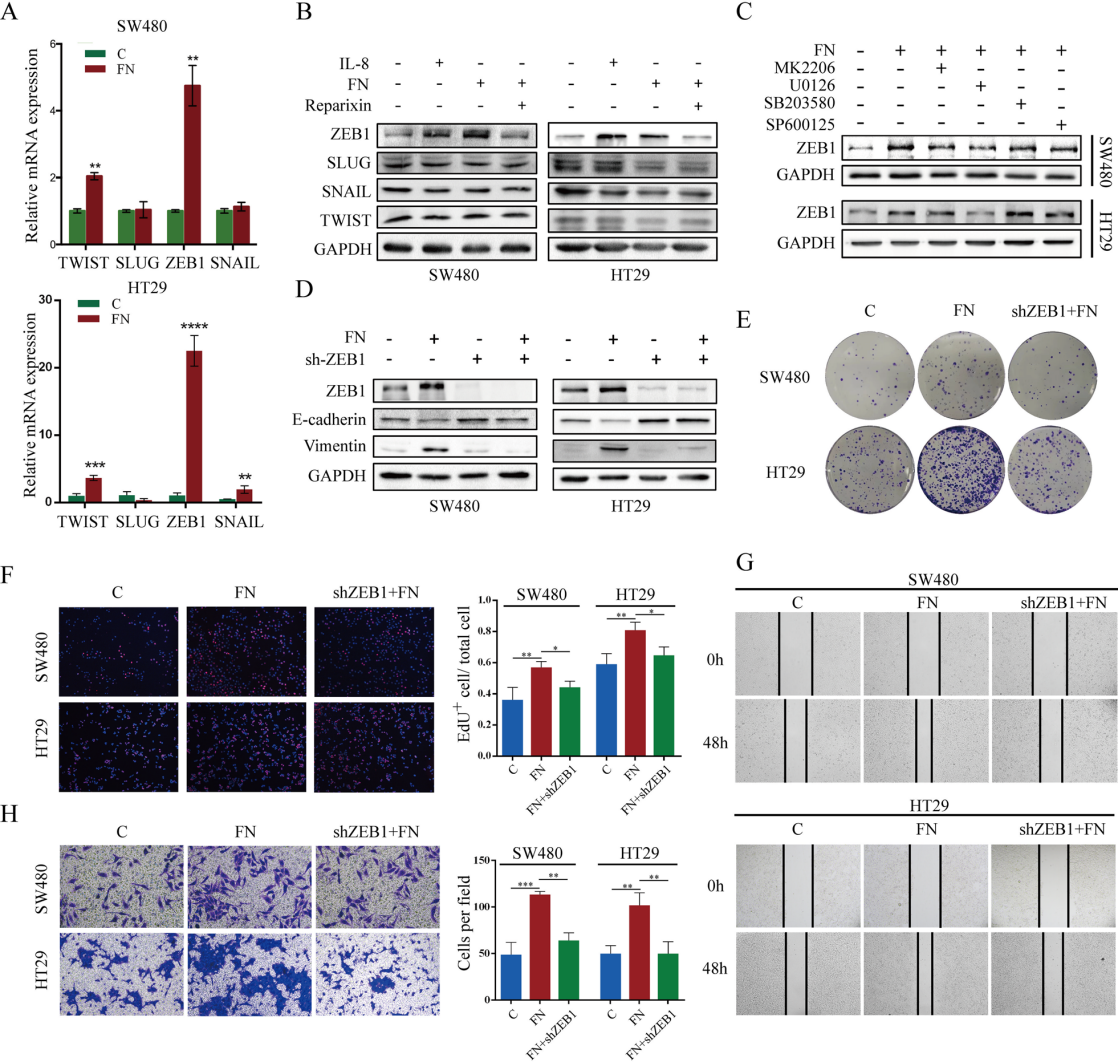
*F. nucleatum* induced IL-8 promotes CRC metastasis through ERK/ZEB1 axis. **A, B** qRT-PCR and western blot analysis of EMT-TFs mRNA and protein level in SW480 and HT29 cells after *F. nucleatum* infection. The Western blot is quantified in Supplementary Figure S2F. **C** *F. nucleatum* increased ZEB1 expression via ERK pathway. The Western blot is quantified in Supplementary Figure S2G. **D** downregulating ZEB1 inhibited the promotion of EMT of CRC cells by *F. nucleatum*. The Western blot is quantified in Supplementary Figure S2H. **E, F** colony formation and EdU assays showed the proliferation capacity of CRC cells. **G, H** wound healing and transwell analysis showed the migration and invasion capacity of CRC cells

The occurrence of EMT is regulated by EMT-related transcription factors (EMT-TFs), such as ZEB1, Snail, Slug and Twist. qRT‒PCR and Western blot assays were also conducted to verify which EMT-TFs are affected by *F. nucleatum* in colorectal cancer cells. In the IL-8 treated group and *F. nucleatum* coculture group, ZEB1 expression was significantly increased compared to control, while the increase in ZEB1 expression induced by *F. nucleatum* was reversed after treatment with the IL-8 receptor inhibitor Reparixin (Fig. 7A, B), indicating that *F. nucleatum* can promote ZEB1 expression in colorectal cancer cells via the IL-8 pathway. Moreover, the promotion of ZEB1 expression in colorectal cancer cells by *F. nucleatum* was dependent on the ERK signaling pathway (Fig. 7C). *F. nucleatum* failed to induce EMT in colorectal cancer cells after we knocked down ZEB1 expression by lentivirus transduction (Fig. 7D). Knockdown of ZEB1 inhibited the *F. nucleatum*-mediated promotion of proliferation, migration and invasion in colorectal cancer cells (Fig. 7E–H). These results suggest that *F. nucleatum* regulates the expression of ZEB1 by mediating the activation of the ERK pathway in colorectal cancer cells, thus promoting the metastasis of colorectal cancer.

## Discussion

The metastasis of colorectal cancer continues to pose a significant challenge following curative treatment and represents a crucial factor in CRC-related mortality. Although a growing body of evidence suggests a correlation between the abundance of *F. nucleatum* and CRC metastasis, the molecular mechanisms involved remain elusive [24]. Consequently, it is imperative to comprehend the underlying molecular mechanisms to identify potential targets for inhibiting CRC metastasis. In this study, we found that *F. nucleatum* was highly abundant in the feces of CRC patients and was associated with CRC liver metastasis. We present evidence demonstrating that *F. nucleatum* enhances the migration and invasion of CRC cells by increasing the expression of IL-8 and inducing epithelial–mesenchymal transition.

In order to identify the mechanism by which *F. nucleatum* facilitates CRC metastasis, we performed RNA sequencing (RNA-seq) analysis. By overlapping our RNA-sequencing results and the GEO dataset, we identified IL-8 as a downstream target associated with *F. nucleatum* infection and CRC metastasis. Emerging research has substantiated the crucial role of IL-8 as an inflammatory and immunosuppressive factor within the tumor microenvironment that facilitates tumor progression [18, 25]. Furthermore, increased serum levels of IL-8 have been correlated with poor prognosis in CRC patients [26]. We found that inhibiting the receptor of IL-8 decreased the promotion of *F. nucleatum*-induced CRC proliferation and metastasis in vitro and in vivo, indicating that IL-8 can act as a target for preventing CRC metastasis. In this study, we investigated how *F. nucleatum* induces CRC cells to secrete IL-8, thereby enhancing their invasive capacity and promoting metastasis. Additionally, IL-8 acts as a chemoattractant for immune cells, influencing the immune microenvironment [27]. Our results indicate that *F. nucleatum*-induced IL-8 leads to increased infiltration of macrophages, particularly M2 macrophages, within the tumor. However, the role and mechanisms by which these macrophages, attracted by *F. nucleatum*-induced IL-8, contribute to CRC metastasis warrant further investigation.

To reveal the mechanism by which *F. nucleatum* enhances IL-8 expression in CRC cells, bioinformatics analysis was conducted to predict a series of miRNAs that act as upstream regulators of IL-8. miRNAs can bind to the 3’UTR region of mRNA, causing degradation of the target mRNA [28]. Among these miRNAs, miR-5692a exhibited a negative correlation with *F. nucleatum* infection and IL-8 expression. Our findings demonstrated that *F. nucleatum* modulates the expression of IL-8 through the involvement of miR-5692a. Previous studies have indicated that miR-5692a is upregulated in hepatocellular carcinoma patients and contributes to the malignant progression of HCC by regulating MMP9^29^. In contrast, our results suggest that miR-5692a can suppress CRC metastasis through inhibiting IL-8 expression. It will be of great interest to establish the biological importance of miR-5692 in other cancers.

Previous research has extensively validated the signaling pathways implicated in the progression of tumors, specifically the ERK, p-38, and JNK pathway [30–32]. Our findings show that *F. nucleatum*-induced IL-8 significantly activates the ERK, p-38, and JNK pathways in CRC cells, with the ERK pathway being particularly crucial for promoting EMT. This observation aligns with previous studies that have shown IL-8 as a key mediator in CRC progression through the activation of these pathways [33, 34]. Although studies have reported that the p-38 and JNK pathways can influence EMT in tumor cells [35, 36], our research indicates that the EMT induced by *F. nucleatum* in CRC cells primarily depends on the ERK pathway, rather than the p-38 or JNK pathways. Western blot analysis confirmed that *F. nucleatum*-induced IL-8 expression leads to the phosphorylation of ERK, which in turn elevates ZEB1 expression and promotes EMT in CRC cells. Furthermore, the use of the ERK inhibitor effectively reversed EMT in these cells, underscoring that ERK is the key downstream target of *F. nucleatum*-induced IL-8. By integrating these findings with established literature, our study provides new insights into the mechanisms by which *F. nucleatum-induced* IL-8 promotes CRC progression.

The findings of our study demonstrate the substantial involvement of *F. nucleatum* in the process of CRC liver metastasis. Consequently, the administration of antibiotics may be considered as a viable therapeutic approach for CRC patients exhibiting an abundance of *F. nucleatum*, with the aim of eradicating *F. nucleatum* and mitigating the occurrence of liver metastases. Moreover, the impact of *F. nucleatum* on the proliferation and metastasis of CRC is primarily mediated through the modulation of IL-8 expression. The significant role of IL-8 in preventing *F. nucleatum*-induced CRC liver metastasis was found, which will facilitate future research endeavors aimed at unraveling the underlying mechanisms of CRC metastasis.

However, data from human specimens are lacking to show that aberrant enrichment of *F. nucleatum* leads to increased expression of IL-8. The results need to be confirmed in human specimens to determine additional implications. In the future, we will collect specimens from CRC patients to verify the regulatory relationship between *F. nucleatum* and IL-8 in CRC liver metastasis. In our study, we demonstrated that *F. nucleatum* modulates the expression of IL-8 through miR-5692a. Whereas, the mechanism by which *F. nucleatum* downregulates the expression of miR-5692a has not been determined. In this study, we propose that *Fusobacterium nucleatum*-induced IL-8 activates the ERK pathway, thereby promoting colorectal cancer metastasis. However, the cell lines we used—HT29 with a BRAF mutation and SW480 and CT26 with KRAS mutations—already exhibit an activated ERK state. Although we have supplemented our findings with relevant experiments using CACO-2 and MC38 cell lines, the mechanism by which *Fusobacterium nucleatum* induces IL-8 production via miR-5692a requires validation in these two cell lines in future studies.

## Conclusion

In conclusion, our research offers compelling evidence that *F. nucleatum* infection promotes CRC liver metastasis through the miR5692a/IL-8 axis and EMT. These findings shed light on the intricate molecular mechanisms underlying the regulatory effect of *F. nucleatum* on IL-8 expression in CRC liver metastasis, thereby emphasizing the potential therapeutic value of targeting *F. nucleatum* and IL-8 to impede the progression of *F. nucleatum*-induced CRC liver metastasis.

## Supplementary Information

Supplementary Material 1.

## Data Availability

The data that support the findings of this study are available from the corresponding author, XL Y, upon reasonable request.

## Abbreviations

CRC: Colorectal cancer
DGE: Differentially expressed genes
EMT: Epithelial-mesenchymal transition
EMT-TFs: EMT-related transcription factors
F. nucleatum: Fusobacterium nucleatum
KEGG: Kyoto encyclopedia of genes and genomes
LDA: Linear discriminant analysis scores
MUT: Mutated
TCGA: The cancer genome Atlas
qRT-PCR: Quantitative reverse transcription polymerase chain reaction
WT: Wildtype

## References

[1] Siegel RL, Miller KD, Wagle NS, Jemal A. Cancer statistics, 2023. CA Cancer J Clin. 2023;73(1):17–48. 36633525 10.3322/caac.21763

[2] Sung H, Ferlay J, Siegel RL, et al. Global Cancer statistics 2020: GLOBOCAN estimates of incidence and Mortality Worldwide for 36 cancers in 185 countries. CA Cancer J Clin. 2021;71(3):209–49. 33538338 10.3322/caac.21660

[3] Siegel RL, Wagle NS, Cercek A, Smith RA, Jemal A. Colorectal cancer statistics, 2023. CA Cancer J Clin. 2023;73(3):233–54. 36856579 10.3322/caac.21772

[4] Overman MJ, McDermott R, Leach JL, et al. Nivolumab in patients with metastatic DNA mismatch repair-deficient or microsatellite instability-high colorectal cancer (CheckMate 142): an open-label, multicentre, phase 2 study. Lancet Oncol. 2017;18(9):1182–91. 28734759 10.1016/S1470-2045(17)30422-9 PMC6207072

[5] Takahashi H, Berber E. Role of thermal ablation in the management of colorectal liver metastasis. Hepatobiliary Surg Nutr. 2020;9(1):49–58. 32140478 10.21037/hbsn.2019.06.08 PMC7026789

[6] Al Bandar MH, Kim NK. Current status and future perspectives on treatment of liver metastasis in colorectal cancer (review). Oncol Rep. 2017;37(5):2553–64. 28350137 10.3892/or.2017.5531

[7] Villard C, Habib M, Nordenvall C, Nilsson PJ, Jorns C, Sparrelid E. Conversion therapy in patients with colorectal liver metastases. Eur J Surg Oncol. 2021;47(8):2038–45. 33640172 10.1016/j.ejso.2021.02.019

[8] White MT, Sears CL. The microbial landscape of colorectal cancer. Nat Rev Microbiol. 2023;22:240. 37794172 10.1038/s41579-023-00973-4

[9] Wang Z, Dan W, Zhang N, Fang J, Yang Y. Colorectal cancer and gut microbiota studies in China. Gut Microbes. 2023;15(1):2236364. 37482657 10.1080/19490976.2023.2236364 PMC10364665

[10] Qu R, Zhang Y, Ma Y, et al. Role of the gut microbiota and its metabolites in tumorigenesis or development of Colorectal Cancer. Adv Sci (Weinh). 2023;10(23):e2205563. 37263983 10.1002/advs.202205563 PMC10427379

[11] Cavallucci V, Palucci I, Fidaleo M, et al. Proinflammatory and cancer-promoting pathobiont fusobacterium nucleatum directly targets colorectal cancer stem cells. Biomolecules. 2022;12(9):1256. 36139097 10.3390/biom12091256 PMC9496236

[12] Rubinstein MR, Wang X, Liu W, Hao Y, Cai G, Han YW. Fusobacterium nucleatum promotes colorectal carcinogenesis by modulating E-cadherin/beta-catenin signaling via its FadA Adhesin. Cell Host Microbe. 2013;14(2):195–206. 23954158 10.1016/j.chom.2013.07.012 PMC3770529

[13] Xu C, Fan L, Lin Y et al. Fusobacterium nucleatum promotes colorectal cancer metastasis through miR-1322/CCL20 axis and M2 polarization. Gut Microbes. 2021;13(1):1980347. 34632963 10.1080/19490976.2021.1980347 PMC8510564

[14] Wang N, Fang JY. Fusobacterium nucleatum, a key pathogenic factor and microbial biomarker for colorectal cancer. Trends Microbiol. 2023;31(2):159–72. 36058786 10.1016/j.tim.2022.08.010

[15] Zhang Y, Zhang L, Zheng S, et al. Fusobacterium nucleatum promotes colorectal cancer cells adhesion to endothelial cells and facilitates extravasation and metastasis by inducing ALPK1/NF-κB/ICAM1 axis. Gut Microbes. 2022;14(1):2038852. 35220887 10.1080/19490976.2022.2038852 PMC8890384

[16] Bullman S, Pedamallu CS, Sicinska E, et al. Analysis of Fusobacterium persistence and antibiotic response in colorectal cancer. Science. 2017;358(6369):1443–8. 29170280 10.1126/science.aal5240 PMC5823247

[17] Bhat AA, Nisar S, Singh M, et al. Cytokine- and chemokine-induced inflammatory colorectal tumor microenvironment: emerging avenue for targeted therapy. Cancer Commun (Lond). 2022;42(8):689–715. 35791509 10.1002/cac2.12295 PMC9395317

[18] Shen CJ, Chan RH, Lin BW, et al. Oleic acid-induced metastasis of KRAS/p53-mutant colorectal cancer relies on concurrent KRAS activation and IL-8 expression bypassing EGFR activation. Theranostics. 2023;13(13):4650–66. 37649607 10.7150/thno.85855 PMC10465226

[19] Guo M, Lian J, Liu Y, et al. Loss of miR-637 promotes cancer cell stemness via WASH/IL-8 pathway and serves as a novel prognostic marker in esophageal squamous cell carcinoma. Biomark Res. 2022;10(1):77. 36329557 10.1186/s40364-022-00424-x PMC9635169

[20] Casasanta MA, Yoo CC, Udayasuryan B, et al. Fusobacterium nucleatum host-cell binding and invasion induces IL-8 and CXCL1 secretion that drives colorectal cancer cell migration. Sci Signal. 2020;13:641. 10.1126/scisignal.aba9157 PMC7454160 32694172

[21] Fontana R, Mestre-Farrera A, Yang J. Update on epithelial-mesenchymal plasticity in Cancer Progression. Annu Rev Pathol. 2024;19:133–56. 37758242 10.1146/annurev-pathmechdis-051222-122423 PMC10872224

[22] Nie F, Zhang J, Tian H, et al. The role of CXCL2-mediated crosstalk between tumor cells and macrophages in Fusobacterium nucleatum-promoted oral squamous cell carcinoma progression. Cell Death Dis. 2024;15(4):277. 38637499 10.1038/s41419-024-06640-7 PMC11026399

[23] Nomoto D, Baba Y, Liu Y, et al. Fusobacterium nucleatum promotes esophageal squamous cell carcinoma progression via the NOD1/RIPK2/NF-κB pathway. Cancer Lett. 2022;530:59–67. 35033591 10.1016/j.canlet.2022.01.014

[24] Yan X, Liu L, Li H, Qin H, Sun Z. Clinical significance of Fusobacterium nucleatum, epithelial-mesenchymal transition, and cancer stem cell markers in stage III/IV colorectal cancer patients. Onco Targets Ther. 2017;10:5031–46. 29081665 10.2147/OTT.S145949 PMC5652912

[25] Lopez-Bujanda ZA, Haffner MC, Chaimowitz MG, et al. Castration-mediated IL-8 promotes myeloid infiltration and prostate cancer progression. Nat Cancer. 2021;2(8):803–18. 35122025 10.1038/s43018-021-00227-3 PMC9169571

[26] Ogawa R, Yamamoto T, Hirai H, et al. Loss of SMAD4 promotes colorectal Cancer progression by recruiting tumor-associated neutrophils via the CXCL1/8-CXCR2 Axis. Clin Cancer Res. 2019;25(9):2887–99. 30705034 10.1158/1078-0432.CCR-18-3684

[27] Gu X, Zhu Y, Su J, et al. Lactate-induced activation of tumor-associated fibroblasts and IL-8-mediated macrophage recruitment promote lung cancer progression. Redox Biol. 2024;74:103209. 38861833 10.1016/j.redox.2024.103209 PMC11215341

[28] Fabian MR, Sonenberg N. The mechanics of miRNA-mediated gene silencing: a look under the hood of miRISC. Nat Struct Mol Biol. 2012;19(6):586–93. 22664986 10.1038/nsmb.2296

[29] Sun SJ, Wang N, Sun ZW, Chen J, Cui HW. MiR-5692a promotes the invasion and metastasis of hepatocellular carcinoma via MMP9. Eur Rev Med Pharmacol Sci. 2018;22(15):4869–78. 30070322 10.26355/eurrev_201808_15623

[30] Maharati A, Moghbeli M. PI3K/AKT signaling pathway as a critical regulator of epithelial-mesenchymal transition in colorectal tumor cells. Cell Commun Signal. 2023;21(1):201. 37580737 10.1186/s12964-023-01225-x PMC10424373

[31] Stefani C, Miricescu D, Stanescu S, et al. Growth factors, PI3K/AKT/mTOR and MAPK signaling pathways in Colorectal Cancer Pathogenesis: where are we now? Int J Mol Sci. 2021;22:19. 10.3390/ijms221910260 PMC8508474 34638601

[32] Chen C, Liu Y, Liu L, et al. Exosomal circTUBGCP4 promotes vascular endothelial cell tipping and colorectal cancer metastasis by activating akt signaling pathway. J Exp Clin Cancer Res. 2023;42(1):46. 36793126 10.1186/s13046-023-02619-y PMC9930311

[33] Lou M, Iwatsuki M, Wu X, Zhang W, Matsumoto C, Baba H. Cancer-associated fibroblast-derived IL-8 upregulates PD-L1 expression in gastric Cancer through the NF-κB pathway. Ann Surg Oncol. 2024;31(5):2983–95. 38006530 10.1245/s10434-023-14586-x

[34] Zhou Q, Jin P, Liu J, Li S, Liu W, Xi S. HER2 overexpression triggers the IL-8 to promote arsenic-induced EMT and stem cell-like phenotypes in human bladder epithelial cells. Ecotoxicol Environ Saf. 2021;208:111693. 33396024 10.1016/j.ecoenv.2020.111693

[35] Sun PH, Xia S, Yuan R, Zhang B, Wang G. TMEM176B promotes EMT via FGFR/JNK signalling in development and tumourigenesis of lung adenocarcinoma. Cancers (Basel). 2024;16(13):2447. 39001509 10.3390/cancers16132447 PMC11240709

[36] Yan W, Wang X, Wang W et al. The p38/MAPK pathway as a therapeutic target to prevent therapeutic escape of breast cancer stem cells. Sci China Life Sci. 2024. 38951428 10.1007/s11427-023-2585-5

