# Supplementary material for: Fusobacterium nucleatum promotes colorectal cancer liver metastasis via miR-5692a/IL-8 axis by inducing epithelial-mesenchymal transition
Source: J Biomed Sci. 2025 Jan 6;32:5. doi: 10.1186/s12929-024-01097-4 (PMC11702224; doi:10.1186/s12929-024-01097-4)
Supplement: Supplementary file 1 — Supplementary Material 1. [file 12929_2024_1097_MOESM1_ESM.docx]

**Table S1 Primer sequence**

| Primer name | | Primer sequences |
| --- | --- | --- |
| *GAPDH*-Forward primer | 5´-GTGAAGGTCGGAGTCAACGG-3´ | |
| *GAPDH*-Reverse primer | 5´-CCTGGAAGATGGTGATGGGAT-3´ | |
| *E-CADHERIN*-Forward primer | 5´-CGAGAGCTACACGTTCACGG-3´ | |
| *E-CADHERIN*-Reverse primer | 5´-GGGTGTCGAGGGAAAAATAGG-3´ | |
| *VIMENTIN*-Forward primer | 5´-GACGCCATCAACACCGAGTT-3´ | |
| *VIMENTIN*-Reverse primer | 5´-CTTTGTCGTTGGTTAGCTGGT-3´ | |
| *ZEB1*-Forward primer | 5´-GATGATGAATGCGAGTCAGATGC-3´ | |
| *ZEB1*-Reverse primer | 5´-ACAGCAGTGTCTTGTTGTTGT-3´ | |
| *IL8*-Forward primer | 5'-ACTCCAAACCTTTCCACC-3' | |
| *IL8*-Reverse primer | 5'-CTTCTCCACAACCCTCTG-3' | |
| *SLUG*-Forward primer | 5´-CGAACTGGACACACATACAGTG-3´ | |
| *SLUG*-Reverse primer | 5´-CTGAGGATCTCTGGTTGTGGT-3´ | |
| *SNAIL1*-Forward primer | 5´-TCGGAAGCCTAACTACAGCGA-3´ | |
| *SNAIL1*-Reverse primer | 5´-AGATGAGCATTGGCAGCGAG-3´ | |
| *TWIST1*-Forward primer | 5´-GTACATCGACTTCCTCTACCAG-3´ | |
| *TWIST1*-Reverse primer | 5´-CATCCTCCAGACCGAGAAG-3´ | |

| Antibody | Species | Company | Catalog | Dilution |
| --- | --- | --- | --- | --- |
| anti-GAPDH | mouse | Proteintech | 60004-1-Ig | 1:5000 |
| anti-E-cadherin | mouse | Abcam | 3195 | 1:1000 |
| anti-Vimentin | mouse | Proteintech | 10366-1-AP | 1:1000 |
| anti-Akt | rabbit | CST | 4685 | 1:1000 |
| anti-p-Akt | rabbit | CST | 4060 | 1:1000 |
| anti-Erk | rabbit | Abclonal | A4782 | 1:1000 |
| anti-p-Erk | rabbit | Abclonal | AP0485 | 1:1000 |
| anti-p38 | rabbit | CST | 9212 | 1:1000 |
| anti-p-p38 | rabbit | CST | 9215 | 1:1000 |
| anti-JNK | rabbit | CST | 9252 | 1:1000 |
| anti-p-JNK | rabbit | CST | 4668 | 1:1000 |
| anti-IL8 | rabbit | CST | 94407 | 1:1000 |
| anti-ZEB1 | rabbit | CST | 70512 | 1:1000 |
| anti-Snail | rabbit | Proteintech | 13099-1-AP | 1:1000 |
| anti-Slug | rabbit | Proteintech | 12129-1-AP | 1:1000 |
| anti-Twist1 | rabbit | Proteintech | 25465-1-AP | 1:1000 |

**Table S2 The primary antibodies**


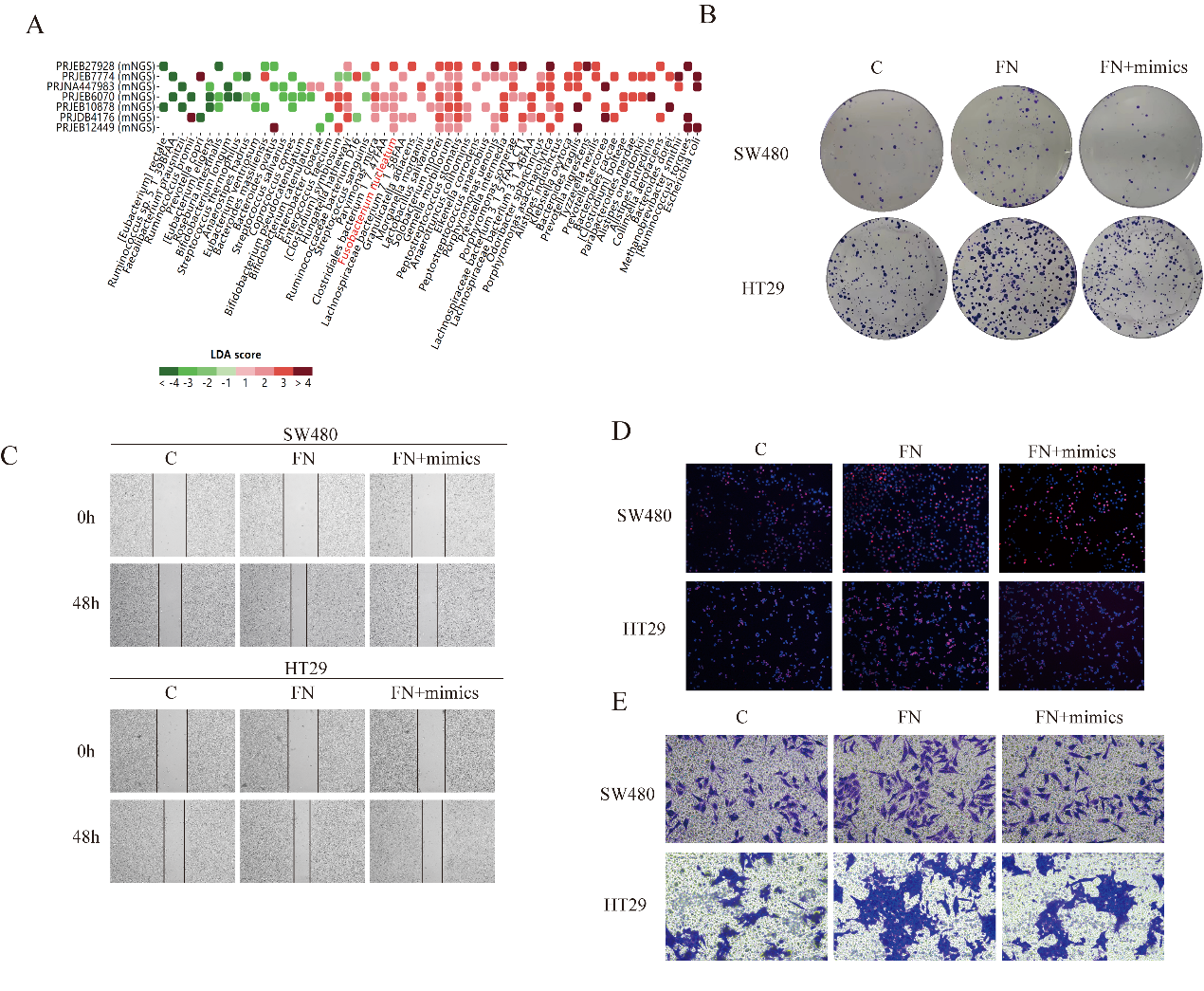


Figure S1 (A) the marker taxa in CRC patients. (B) colony formation showed the proliferation capacity of CRC cells treated with *F. nucleatum* and/or mimics. (C)EdU assays showed the proliferation capacity of CRC cells treated with *F. nucleatum* and/or mimics. (D, E) wound healing and transwell analysis showed the migration and invasion capacity of CRC cells treated with *F. nucleatum* and/or mimics.


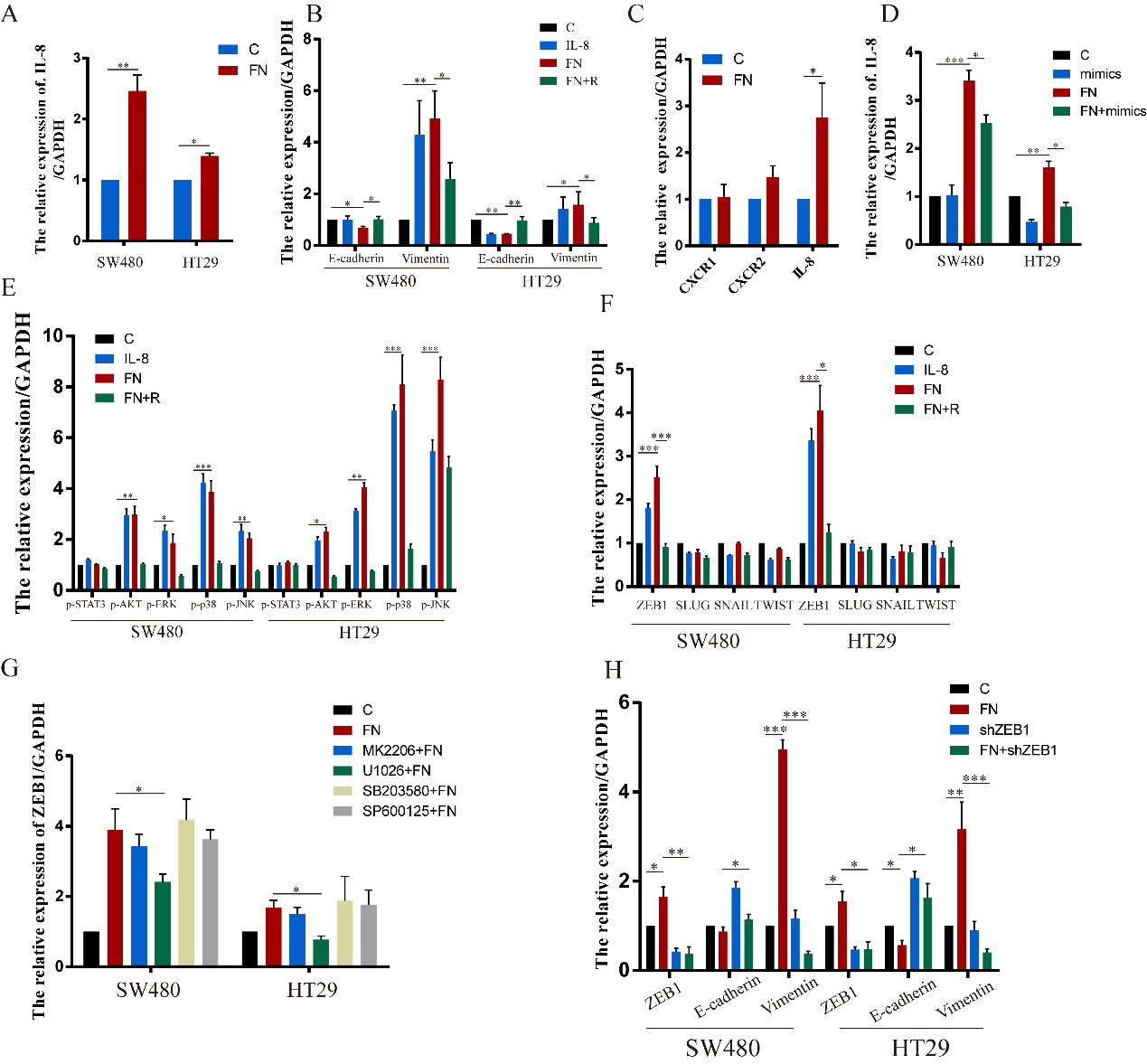


Figure S2 The quantitative data of the western blot results. (A) Quantification for the Western blot shown in Figure 2E. (B) Quantification for the Western blot shown in Figure 3E. (C) Quantification for the Western blot shown in Figure 4F. (D) Quantification for the Western blot shown in Figure 5F. (E) Quantification for the Western blot shown in Figure 6B. (F) Quantification for the Western blot shown in Figure 7B. (G) Quantification for the Western blot shown in Figure 7C. (H) Quantification for the Western blot shown in Figure 7D.


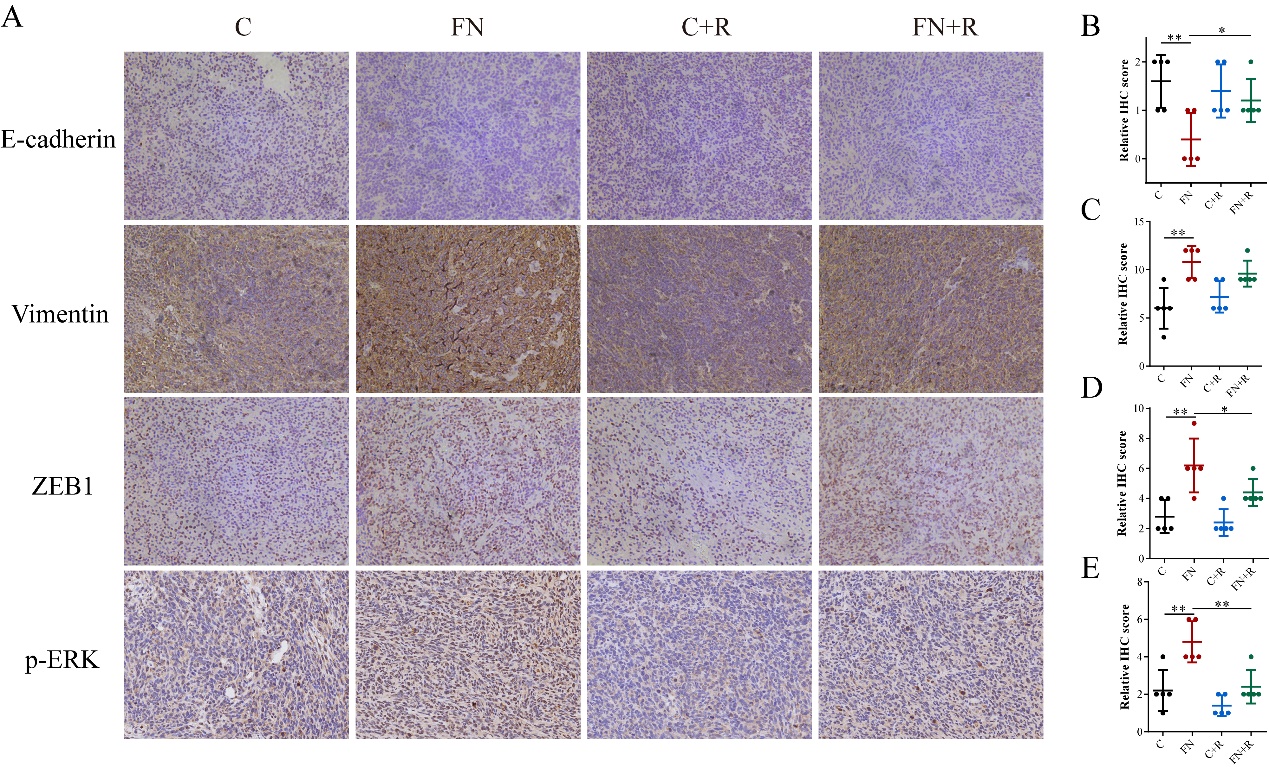


Figure S3 (A) The immunohistochemistry results of E-cadherin, vimentin, ZEB1, and p-ERK in xenograft tumor sections. (B) the relative IHC scores of E-cadherin. (C) the relative IHC scores of Vimentin. (D) the relative IHC scores of ZEB1. (E) the relative IHC scores of p-ERK. The relative expression was scored according to the staining scope and intensity. Specifically, the staining scope was: 1 (0–25%); 2 (25–50%); 3 (50–75%); and 4 (75–100%), and the staining intensity was scored as 0 (negative); 1 (weakly positive); 2 (moderately positive); and 3 (strongly positive). The overall score was defined by multiplying the staining scope by the staining intensity score.


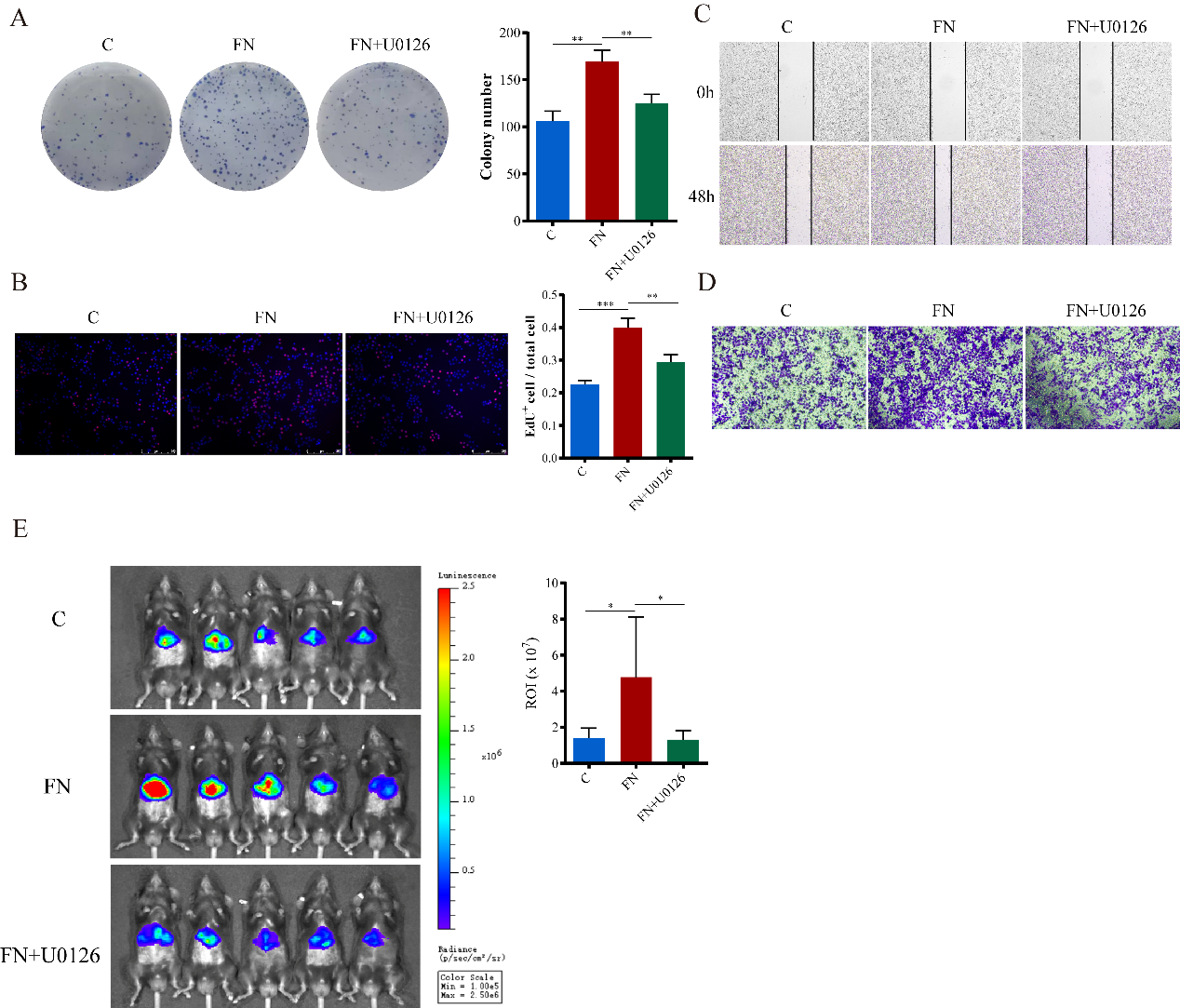


Figure S4 (A)colony formation and (B) EdU assays showed the proliferation capacity of CACO-2 cells treated with F. nucleatum and/or U0126. (C) wound healing and (D) transwell analysis showed the migration and invasion capacity of CACO-2 cells treated with F. nucleatum and/or U0126. (E) The liver metastases of MC38 cells were assessed by in vivo bioluminescence imaging.
